# Consideration of Ketogenic Metabolic Therapy as a Complementary or Alternative Approach for Managing Breast Cancer

**DOI:** 10.3389/fnut.2020.00021

**Published:** 2020-03-11

**Authors:** Thomas N. Seyfried, Purna Mukherjee, Mehmet S. Iyikesici, Abdul Slocum, Miriam Kalamian, Jean-Pierre Spinosa, Christos Chinopoulos

**Affiliations:** ^1^Biology Department, Boston College, Chestnut Hill, MA, United States; ^2^Medical Oncology, Kemerburgaz University Bahcelievler Medical Park Hospital, Istanbul, Turkey; ^3^Medical Oncology, Chemo Thermia Oncology Center, Istanbul, Turkey; ^4^Dietary Therapies LLc, Hamilton, MT, United States; ^5^Gynecologic and Breast Oncology, Clinique Cecil, Lausanne, Switzerland; ^6^Department of Medical Biochemistry, Semmelweis University, Budapest, Hungary

**Keywords:** glycolysis, survival, glutaminolysis, non-toxic, fermentation, metastasis, inflammation

## Abstract

Breast cancer remains as a significant cause of morbidity and mortality in women. Ultrastructural and biochemical evidence from breast biopsy tissue and cancer cells shows mitochondrial abnormalities that are incompatible with energy production through oxidative phosphorylation (OxPhos). Consequently, breast cancer, like most cancers, will become more reliant on substrate level phosphorylation (fermentation) than on oxidative phosphorylation (OxPhos) for growth consistent with the mitochondrial metabolic theory of cancer. Glucose and glutamine are the prime fermentable fuels that underlie therapy resistance and drive breast cancer growth through substrate level phosphorylation (SLP) in both the cytoplasm (Warburg effect) and the mitochondria (Q-effect), respectively. Emerging evidence indicates that ketogenic metabolic therapy (KMT) can reduce glucose availability to tumor cells while simultaneously elevating ketone bodies, a non-fermentable metabolic fuel. It is suggested that KMT would be most effective when used together with glutamine targeting. Information is reviewed for suggesting how KMT could reduce systemic inflammation and target tumor cells without causing damage to normal cells. Implementation of KMT in the clinic could improve progression free and overall survival for patients with breast cancer.

## Introduction

Breast cancer persists as a significant cause of morbidity and mortality in woman. According to the American Cancer Society, the number of new cases and deaths from breast cancer in US woman is estimated to be 268,600 and 41,760, respectively, for 2019 (1). Indeed, breast cancer alone will account for 30% of all female cancers. Although the incidence of breast cancer is lower in black women than in white women, the death rate is 41% higher in blacks than in whites possibly due in part to diet and lifestyle (2). The failure to effectively manage malignant breast cancer, and most malignant cancers for that matter, comes in large part from a misunderstanding on the origin of cancer. Although cancer has long been considered a genetic disease based on the somatic mutation theory (3–5), recent revelations have raised serious concerns that question the validity of this theory. Major concerns include:

1 The absence of gene and chromosomal mutations in some cancers (6–9). Indeed, Greenman et al. found no mutations following extensive sequencing in 73/210 cancers (3), while Parsons et al. found no mutations in the P53, the PI3K, and the RB1 pathways in the Br20P tissue sample of a glioblastoma patient (10). Such samples should not exist according to the somatic mutation theory.
2 The presence and clonal expansion of numerous so-called driver gene mutations in a broad range of normal human tissues including breast tissue (11–15). It is not clear how the somatic mutation theory can account for malignant tumors that have no mutations or for normal cells and tissues that express driver mutations.
3 The absence of breast cancer and most other cancers in chimpanzees despite having about 98.5% gene and protein sequence identity with humans even at the BRCA1 locus (16–19). Indeed, breast cancer has never been documented in a female chimpanzee suggesting that diet and lifestyle issues, rather than genetic mutations, are largely responsible for the disease (18, 20).
4 The nuclear/cytoplasm transfer experiments showing that normal cells and tissues can be produced from tumorigenic nuclei as long as the tumorigenic nuclei are localized in cytoplasm containing normal mitochondria (21). Furthermore, recent studies show that normal mitochondria can down-regulate multiple oncogenic pathways and growth behavior in metastatic breast cancer cells (22, 23). These findings show that normal mitochondrial function can suppress tumorigenesis regardless of the gene or chromosomal abnormalities that might be present in the tumor nucleus. Viewed collectively, these findings suggest that the somatic mutations found in breast cancer and in most other cancers are not the primary cause of the disease. It is therefore unlikely that therapeutic strategies based on the somatic mutation theory will have major impact on the management of most cancers including breast cancer.

## The Mitochondrial Metabolic Theory of Cancer

**Figure 1:**
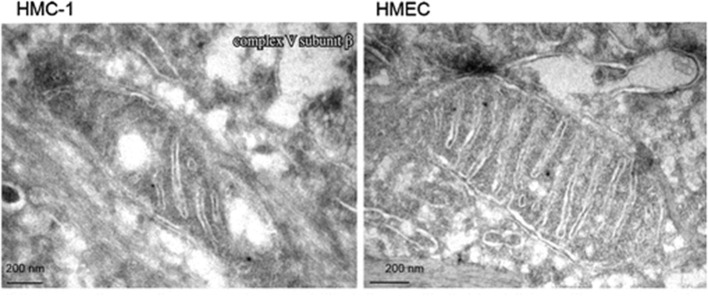
Electron microscopy of primary breast cancer cells (human mammary carcinoma HMC-1) and human epithelial mammary cell control line (HEMC). Abnormal mitochondrial morphology in the HMC-1 cell showing loss of invaginations and vacuoles. These abnormalities in mitochondria ultrastructure were linked to abnormalities in the electron transport chain and are in general agreement with those from other studies of breast cancer mitochondria (37, 40, 42). Reprinted with permission from Putignani et al. (43).

Emerging evidence indicates that most if not all cancers display deranged energy metabolism (24–35). It is well-documented that the tumor cells found in most cancerous tissues including breast cancer tissue, have abnormalities in the number, structure, and function of their mitochondria (26, 27, 29, 33, 36–42). These abnormalities would compromise efficient energy production through oxidative phosphorylation (OxPhos). Figure 1 documents ultrastructural abnormalities in breast cancer mitochondria that are linked to abnormalities in proteins of the electron transport chain (43). In addition to abnormalities in mitochondrial membranes, breast cancer cells also express abnormalities in mitochondrial-associated membranes (MAM), that would further reduce energy production through OxPhos (44, 45). Consequently, increased fermentation metabolism would be necessary to compensate for OxPhos deficiency in order to maintain sufficient energy for breast cancer viability and growth.

## Aerobic Fermentation of Glucose and Glutamine in Cancer Cells

Glucose and glutamine are the major fermentable fuels used by cancer cells with impaired OxPhos (39, 46). Glucose is fermented to lactic acid through glycolysis in the cell cytoplasm, while glutamine is fermented to succinic acid through glutaminolysis in the tricarboxylic acid (TCA) cycle. Notably, both fermentation processes can generate energy in the presence or absence of oxygen through substrate level phosphorylation (SLP) at the pyruvate kinase reaction in glycolysis and at the succinate ligase reaction in glutaminolysis, respectively (46). Warburg first described the aerobic fermentation of glucose as a major phenotype of most cancers (47–51). This metabolic phenotype has become known as the Warburg effect (52). In contrast to the Pasteur effect, where fermentation is suppressed in the presence of oxygen, the Warburg effect involves robust glucose-derived lactic acid fermentation even in the presence of 100% oxygen (aerobic fermentation).

## Glutaminolysis

**Figure 2:**
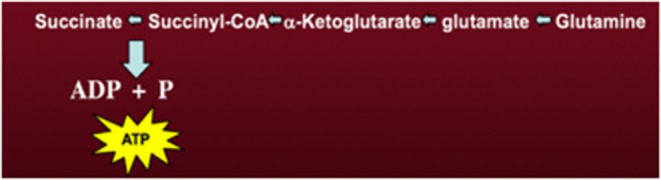
The glutaminolysis pathway. The succinyl-CoA ligase reaction, metabolizing succinyl-CoA to succinate, produces high-energy phosphates (ATP) in the absence of oxidative phosphorylation through the process of substrate-level phosphorylation in the mitochondrial matrix. Provision of succinyl-CoA by the α-ketoglutarate dehydrogenase complex is crucial for maintaining the function of succinyl-CoA ligase thus preventing the adenine nucleotide translocase from reversing. Succinate contributes to inflammation and stabilizes Hif-1a, a key transcription factor that contributes to the aerobic fermentation (53–56).

In addition to aerobic fermentation in the cytoplasm, glutaminolysis can also support high-energy phosphate synthesis in the mitochondria through the sequential conversion of glutamine-glutamate-*alpha*-ketoglutarate-succinyl CoA-succinate (Figure 2) (46). ATP synthesis through the succinate-CoA ligase reaction in the TCA cycle can compensate for reduced ATP synthesis through either glycolysis or OxPhos. We recently proposed that most of the ATP synthesized in tumor cells would come from mitochondrial substrate level phosphorylation (mSLP) at the succinate ligase reaction (46). Evidence showing that mSLP can compensate for OxPhos deficiency in cancer is emerging (57–60). mSLP could also compensate for minimal energy production through glycolysis due to the predominance of the glycolytic pyruvate kinase M2 (PKM2) isoform, which produces less ATP than the PKM1 isoform (61). The PKM2 isoform is predominant in many cancers including breast cancer (62, 63). As *Q* is the single-letter designation of glutamine, the aerobic fermentation of glutamine through mSLP was recently defined as the *Q effect*; which has been recognized as the missing link in the mitochondrial metabolic theory of cancer (32, 46, 64, 65). Unfortunately, the glutaminolysis pathway and the role of glutamine and mSLP was unknown to Warburg (46). We find it remarkable that nearly all of the major reviews or previous studies on cancer energy metabolism have not addressed or possibly even recognized the role of SUCL activity and mSLP, as a compensatory energy mechanism for deficient OxPhos. We consider mSLP as the key mechanism that underlies energy production in tumor cells with defective respiration (46).

Aerobic fermentation (Warburg effect) is a common metabolic phenotype in breast cancer regardless of histopathological type, grade, or gene expression profile (24, 66–74). In addition to glucose, glutamine is the other major fuel necessary for breast cancer cells (73, 75–78). The elevated utilization of glucose and glutamine becomes necessary to sustain the viability of OxPhos-impaired cancer cells through SLP in the cytoplasm and mitochondria, respectively. The Q-effect provides a rational explanation for the high glutamine use in cells with compromised OxPhos (46). The aerobic fermentation of glucose and glutamine in cancer cells would be the expected consequence of impaired OxPhos, as fermentation can compensate for insufficient respiration. Some have suggested that the function of the Warburg-effect is to provide a growth advantage for tumor cells. This is a teleological explanation, i.e., design with a purpose or intelligent design, which should not be part of modern biological thought (79–81). Cells do not make choices or have preferences, but simply respond to conditions in their internal and external environments according to evolutionarily designed metabolic programs. As proliferation, rather than quiescence, is the default state of metazoan cells (8, 82), unbridled proliferation becomes the consequence when fermentation gradually replaces respiration in cancer cells (21, 83, 84). Indeed, unbridled proliferation was the dominant growth phenotype of all organisms that existed on the planet before oxygen entered the atmosphere some 2.5 billion years ago (83). Hence, glucose and glutamine become the drivers of cancer cell fermentation and growth.

## Role of Reactive Oxygen Species (ROS) and Oncogenes in the Origin and Progression of Cancer

Impaired OxPhos together with compensatory fermentation leads to the accumulation of reactive oxygen species (ROS) (85–87). ROS are carcinogenic and mutagenic, and are largely responsible for the genomic instability and mutations seen in tumor cells (85, 87–93). In other words, the mutations seen in tumor cells arise as a consequence of impaired energy metabolism (32, 64). Oncogenes such as *Hif-1alpha, Myc, Ras, BRAF*, etc., facilitate the dependence of tumor cells on glucose and glutamine while defects in the tumor suppressor genes *p53* and *pRb* will compromise OxPhos function thus causing further dependency on fermentation for growth (28, 46, 84, 94–99). These genes mutations are linked to breast cancer and other cancers through mitochondrial dysfunction. Compromised OxPhos would require compensatory glucose and glutamine fermentation to maintain membrane pump activity and tumor cell viability (64, 100). A mutation in the *p53* gene was found to increase glucose consumption and the Warburg effect, whereas the retinoblastoma Rb protein was found to increase glutamine metabolism through an effect on the E2F-3 transcription factor (101, 102). Mutations in these and in other oncogenes, and tumor suppressor genes, have been detected in breast cancer cells (103, 104). It is interesting that Her2 signaling in breast tumor promotes glycolysis and glucose utilization with lactate accumulation (105). Moreover, the ErbB2 targeting antibody, trastuzumab (Herceptin), inhibits glycolysis via downregulation of HSF1 and LDH-A in ErbB2-positive cancer cells. The results of Ding suggest that ErbB2 not only promotes glycolysis, but also inhibits mitochondrial oxidative phosphorylation by decreasing ETC activities (106). Mutations in the *BRCA1* gene, which is known to play an important role in maintaining metabolic homeostasis, have also been linked to elevated glycolysis and other metabolic disturbances (107, 108). As no known inherited breast cancer gene is 100% penetrant, germline mutations are considered secondary risk factors and cannot therefore be considered primary causes of cancer. A penetrance of 100% is necessary for any tumor gene to be considered a primary cause of cancer. OxPhos impairment with compensatory glucose and glutamine fermentation is the common underlying phenotype of most if not all cancers.

## Origin of Metastasis

Metastasis involves the dissemination of cancer cells from the primary tumor to surrounding tissues and to distant organs and is the primary cause of cancer morbidity and mortality (109). Most tumors that fail to show local invasion or metastasis are considered benign and do not pose serious risk despite expressing a majority of the so called “Hallmarks of Cancer” (110). Tumor cell metastasis involves a stereotypic cascade of biological phenomenon including local invasion, intravasation into the blood, survival in the circulation, immune suppression, extravasation from the blood, and growth in a distant organ or tissue (111). Identification of the unique features of the metastatic cell can expand understanding of metastasis and facilitate rational therapeutic strategies based on knowledge of cell biology and biochemistry.

Two major theories have been advanced to explain metastasis that include the epithelial-mesenchymal transition (EMT), and the macrophage fusion hybrid hypothesis. The EMT is the dominant explanation and is based on the somatic mutation theory of cancer. The EMT proposes that an initial series of random mutations disturb cell-cell interaction causing a normal epithelial cell to transform into an invasive mesenchymal cell. Further additional random mutations cause the mesenchymal cell to intravasate, and subsequently extravasate into the parenchyma at some distant organ site (4, 112). Once established at the distant site, the metastatic tumor cells transform back to an epithelial phenotype via the so called mesenchymal epithelial transition (MET) (4, 112). No explanation has been presented, however, as to how the multiple random gene mutations responsible for the initial events of the metastatic cascade could be reversed or suppressed during the MET (4). Despite its linkage to phenomenology, the EMT is considered the dominant explanation for breast cancer metastasis (113).

In contrast to the EMT, the macrophage fusion hybrid hypothesis posits that metastasis arises from macrophages either directly or indirectly from the fusion of macrophages with neoplastic cells (109, 114–117). The metastatic breast cancer cell, like many metastatic cancer cells, expresses characteristics of myeloid cells and macrophages (109, 114, 118–125). These characteristics include phagocytosis, fusogenicity, and expression of multiple myeloid/macrophage biomarkers. Fusion hybridization could also better account for the large degree of cellular and genetic heterogeneity seen in metastatic breast cancer than can the EMT (126, 127). A macrophage origin of metastasis is also more consistent with Paget's “seed soil” hypothesis than is an origin based on EMT (109, 128, 129). Macrophages (seeds) are known to infiltrate organs (soil) non-randomly (109). As glutamine is a major fuel for macrophages (130, 131), targeting the availability of glutamine together with glucose becomes a rational, yet largely overlooked, therapeutic strategy for managing metastatic cancer (60).

## Current Standard of Care (SOC) for Breast Cancer

Current standard management of breast cancer requires histological diagnosis obtained by core needle biopsy prior to surgical procedure (132). Therapies for breast cancer depend largely on the stage, grade, and the biology of the disease. Surgery and radiotherapy (breast and axilla) are used for local disease control, staging, and tumor extirpation. It is important to mention, however, that the extent of surgery and radiotherapy are not linked to a survival advantage (133). Chemotherapy, hormone therapy (tamoxifen, aromatase inhibitor, or one followed by the other), targeted therapies, trastuzumab (Herceptin) and pertuzumab (Perjeta), or combinations of these are designed to destroy local residual cancer cells and latent metastasis, while also reducing the risk of recurrence. Mechanical interventions (core needle biopsies and surgery) can cause wound-induced inflammatory oncotaxis, which influences the natural history of breast cancer (129). Data from several studies show that biopsies and surgery can cause inflammatory oncotaxis thus increasing tissue angiogenesis and spread of tumor cells (134–140). Invasive and metastatic behavior would be expected for breast cancer cells with myeloid properties (123). Some have hypothesized that growth factor stimulation in response to intraoperative tissue damage, can increase HER2 receptor activation in incompletely resected pre-invasive breast cancer thus increasing risk for tumor cell proliferation and spread (141). Procedures that might elevate blood glucose or insulin levels should be avoided, as glucose is known to accelerate breast cancer development (142–147). Products used in anesthesia might also increase blood glucose and insulin levels (148). The type of anesthesia and analgesia used during and after surgery should be carefully monitored for possible influence on glucose and insulin metabolism (149). Glucocorticoids, which are given to some breast cancer patients, can also elevate blood glucose levels thus supporting glucose-dependent aerobic fermentation. In addition to elevating blood glucose, glucocorticoids can also block estrogen-induced apoptosis to further stimulate breast cancer growth (150). Hence, risk for tumor spread can be associated with accepted procedures used in breast cancer management.

## Ketogenic Metabolic Therapy

Ketogenic metabolic therapy (KMT) is emerging as an effective complementary or alternative therapeutic strategy for managing a broad range of malignant cancers including breast cancer (151–160). Calorie restriction and low-carbohydrate high-fat ketogenic diets (KD) reduce the glucose needed to propell the Warburg effect while also elevating ketone bodies (34). Cancer cells cannot effectively use ketone bodies or fatty acids for ATP synthesis through OxPhos due to defects in the number, structure, and function of their mitochondria (34, 46). Moreover, ketone bodies and fatty acids cannot be fermented, and thus cannot effectively replace glucose and glutamine as an alternative energy source for cancer (46). Bartmann et al. showed that the major ketone body, beta-hydroxybutyrate, could not stimulate breast tumor growth *in vitro* (161). Hence, KMT becomes a putative therapeutic strategy for managing most cancers including breast cancer (34).

There have been reports, however, suggesting that some cancers, including breast cancer, can oxidize ketone bodies and fatty acids for growth (162–165). The uptake of ketone bodies or fatty acids together with oxygen consumption in tumor cells is not proof that the ketone bodies or fatty acids can be used to generate energy through OxPhos (34, 46, 166). Indeed, Kuok et al. recently showed that palmitate could increase oxygen consumption rate (OCR) by stimulating ATP usage and insulin secretion rather than by increasing beta-oxidation (167). Fatty acids are potent swelling and uncoupling agents that can stimulate insulin secretion and glucose/glutamine consumption thus making it appear as if tumor cells can metabolize fatty acids for energy (166, 168–170). In other words, fatty acids can stimulate utilization of glucose and glutamine. Many tumor cells including breast cancer cells will store fatty acids as lipid droplets (166, 171, 172). Lipid droplet storage is considered a protection mechanism from the lethal effects of saturated fatty acids in cells that cannot metabolize fats for energy (171–174). If tumor cells could use fatty acids for growth, then water-only fasting and calorie restricted ketogenic diets should accelerate tumor growth, as these dietary changes elevate free fatty acids in the blood (175, 176). This is clearly not the case. Also, palmitic acid cannot support *in vitro* tumor cell growth in the absence of glucose and glutamine. Any disruption of the mitochondrial proton motive gradient will provide ATP for the F1-F0 ATP synthase thus hydrolyzing ATP rather than synthesizing ATP (46). mSLP will provide ATP for F1-F0-ATP synthase in an effort to maintain a moderate mitochondrial membrane potential and prevent reversal. Based on the foundational biological principle that structure determines function (43, 168, 177, 178), ketone bodies and fatty acids cannot serve as major respiratory fuels for tumor cells containing defects in mitochondrial structure and function (46, 84). Hence, it would be helpful to include evidence of normal mitochondria ultrastructure and electron transport chain activities in reports indicating that ketone bodies and fatty acids are fuels for OxPhos-generated ATP synthesis in cancer cells.

Depletion of fermentable fuels from KMT will facilitate catastrophic tumor cell death. Ketogenic diet appears to work better when used with glutamine targeting and is consumed in restricted amounts (60). The simultaneous targeting of glycolysis and glutaminolysis is now emerging as a potential therapeutic strategy for managing a broad range of cancers including breast cancer (60, 179, 180). KMT reduces circulating levels of glucose and insulin that are needed for rapid tumor growth (176). Excessive consumption of ketogenic diets, however, can provoke tumor growth by causing insulin insensitivity and glucose elevation (176, 181). Hence, KMT becomes a logical therapeutic strategy for managing breast cancer when used correctly.

The microenvironment of many tumors is hypoxic, acidotic, and enriched with glucose and glutamine. Under KMT, this pro-tumorigenic microenvironment becomes less inflamed (84, 182, 183). Restricted KDs and calorie restriction are antiinvasive, antiangiogenic, anti-inflammatory, and capable of killing tumor cells through a pro-apoptotic mechanism (181–188). Metabolism of the major circulating ketone body, D-beta-hydroxybutyrate, reduces reactive oxygen species production through the mitochondrial Co-enzyme Q couple in *normal cells*, while simultaneously elevating oxidative stress in *tumor cells* (34, 189–191). Implementation of KMT prior to any surgical procedure could also benefit patients (152). KMT should decrease the need for dexamethasone pretreatment, a problematic therapy that can inadvertently increase availability of glucose to the tumor cells while also inhibiting chemotherapy-induced apoptosis (192–194). It is well-known that glucose and hyperglycemia contribute to rapid breast cancer growth (146, 195, 196). Consequently, KMT could reduce inflammation and glucose systemically, thus enhancing the anti-tumorigenic properties of the microenvironment.

**Figure 3:**
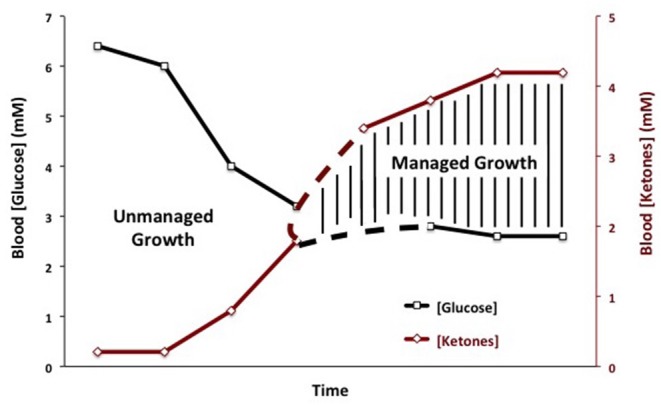
Linkage of plasma glucose and ketone body levels to cancer management. The glucose and ketone (beta-hydroxybutyrate) values are within normal physiological ranges for humans that are under water-only fasting. This state is considered the zone of metabolic management for most cancers. The zone of metabolic management is obtained gradually, as circulating levels of glucose fall and ketones rise. the Glucose Ketone Index (GKI) tracks the transition to therapeutic ketosis. The dashed lines highlight individual variability that could exist in reaching a therapeutic GKI. GKI values approaching 1.0 and below are considered potentially therapeutic. [Reprinted from Meidenbauer et al. (197), and distributed under a Creative Commons license].

Therapeutic ketosis is linked to reduced blood glucose levels and to elevated ketone body levels within normal physiological ranges (Figure 3). Reduction of carbohydrate intake after breast cancer diagnosis in women reduces risk of recurrence (198). Evidence shows that therapeutic ketosis can act synergistically with several drugs and procedures to enhance cancer management while improving both progression-free and overall survival (34, 154, 199, 200). For example, hyperbaric oxygen therapy (HBOT) increases oxidative stress on tumor cells especially when used alongside therapies that reduce blood glucose and elevate blood ketones (201). By reducing blood glucose, KMT would also reduce the immunosuppressive effects of lactic acid in the tumor microenvironment (202). Recent studies show that therapeutic ketosis can facilitate drug delivery through the blood-brain barrier (60, 203). This would be important in helping to target breast cancer cells that metastasize to the brain. Also, it has been reported that caloric restriction reduces leaky tumor blood vessels by increasing neovascular smooth muscle (183). This could account, in part, for a better drug delivery to solid tumors under KMT. The anti-malarial drug, chloroquine neutralizes lysosomal pH reducing phagocytosis and autophagy, and thus depriving invasive and metastatic tumor cells from obtaining glucose and glutamine (34, 204, 205). Chloroquine can also inhibit mitochondrial diaphorases that oxidize NADPH to NAD+, which in turn would reduce mSLP (46, 206). The glutamine dehydrogenase inhibitor, epigallocatechin gallate (EGCG) is also proposed to target glutamine metabolism through an effect on glutamate dehydrogenase (207). The glutaminase inhibitor, 6-diazo-5-oxo-L-norleucine (DON), is a powerful glutamine-targeting drug that can work synergistically with a restricted KD for managing brain cancer and metastasis (60, 208, 209). Hence, KMT can, (a), target the multiple drivers of rapid tumor growth, (b), facilitate drug delivery to the tumor tissue, (c), work synergistically with glutamine-targeting drugs, and, (d), enhance the metabolic efficiency in normal healthy cells. To our knowledge, there are currently no cancer therapies that can target these multiple drivers of tumor growth while simultaneously protecting normal cells.

## The Glucose Ketone Index

**Figure 4:**
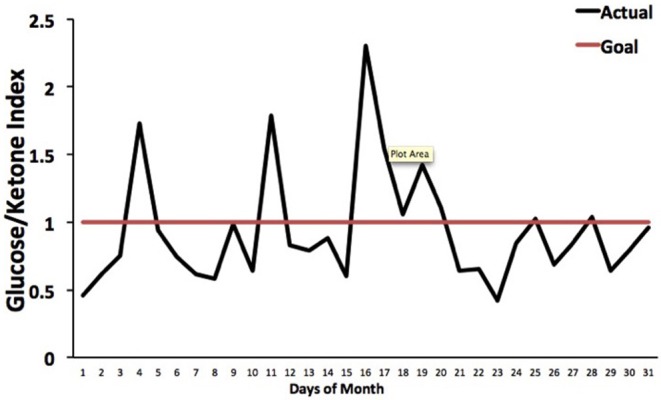
Tracking an individual's GKI using the Glucose Ketone Index Calculator. Index values of 1.0 or below are considered best for managing cancer growth. Individual glucose and ketone values are shown, along with the corresponding GKI values. The GKI values are plotted over the course of a month, whereas the target GKI value (1.0) is plotted as a single line. Tumor management is predicted to be better (slower growth) within the metabolic target zone than outside of the zone. [Reprinted from Meidenbauer et al. (197), and distributed under a Creative Commons license].

The Glucose Ketone Index (GKI) is single number representing the glucose-to-ketone ratio (expressed in mmol/L), and was developed as a guide for evaluating therapeutic efficacy of KMT. The GKI serves as a proxy for the degree of metabolic stress placed on tumor cells through the reduction of circulating glucose and elevation of ketone bodies (beta-hydroxybutyrate, acetoacetate) (34, 197). A GKI value of 1.0 or below has been suggested as the therapeutic goal for cancer management (Figure 4). However, therapeutic GKI values can be difficult to achieve for many cancer patients. For example, tumor burden, toxic treatment protocols, and emotional and physical stress can combine to elevate blood glucose and insulin levels thus preventing the patient from achieving therapeutic GKI values (34, 84, 210). Further refinement of existing KMT therapies along with introduction of new therapies may serve to mitigate this obstacle.

## KMT as a Complementary or Alternative to SOC

**Figure 5:**
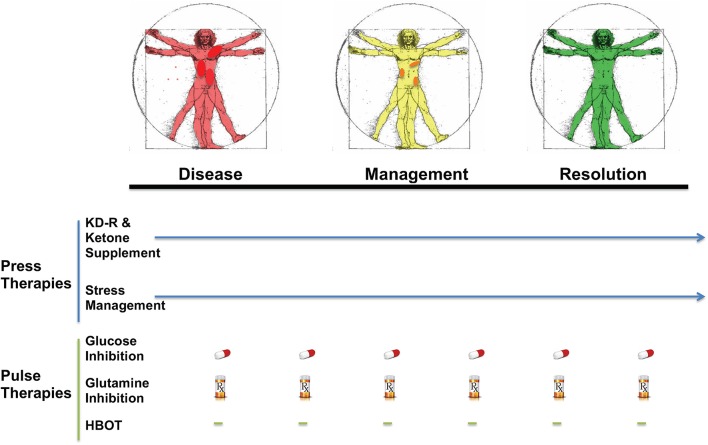
Breast Cancer Management with Press-Pulse Therapeutic Strategy. Arens and West considered the simultaneous occurrence of “Press-Pulse” disturbances as the mechanism responsible for the mass extinction of organic populations during prior evolutionary epochs (213). We described how this concept could be adopted as a therapeutic strategy for the management and possible eradication of cancer (34). This therapeutic strategy considers all cancer, including breast cancer, as a single disease that can be managed by transitioning the energy metabolism of normal cells from glucose to non-fermentable ketone bodies, while simultaneously restricting the availability of fermentable fuels (glucose and glutamine) to tumor cells (34). The reduction in blood glucose levels will also reduce insulin and insulin-like growth factor 1, which are known to drive rapid tumor growth (184, 214, 215). This metabolic therapeutic strategy exploits the dependency of tumor cells on glucose and glutamine fermentation and their inability to metabolize ketone bodies for energy due to defects in the number, structure and function of the tumor mitochondria. In essence, the press-pulse therapy pits the metabolic demands of the mutated tumor cells against those of the normal cells, which evolved to adapt and survive under the extremes of nutrient stress (216). Collections of random mutations will prevent tumor cells from adapting to nutrient stress, thus leading to their extinction according to evolutionary theory (81). As a cancer diagnosis can increase emotional stress and blood glucose, stress management techniques together with exercise could improve general health while reducing glucose availability to the tumor. The press therapies would work synergistically with acute pulse therapies to further restrict glucose and glutamine metabolism. HBOT would work synergistically with the press therapies to increase oxidative stress selectively in tumor cells. The timing (spacing) between the various pulse therapies is designed to stress tumor cell metabolism while minimizing toxicity to normal body cells (34). This therapeutic strategy will target the fermentation metabolism common to most breast tumor cells, thus degrading tumor burden gradually with little or no toxicity. The color change from red (diseased with darker red spots indicative of metastatic lesions), to yellow (with reduced metastasis), to green (resolution) in the Vitruvian man indicates the gradual metabolic management and possible resolution of the breast cancer. The pill and Rx symbols are suggestive of drugs taken orally and/or intravenously (prescription) that would be effective in targeting simultaneously glycolysis and glutaminolysis. Pulse therapies would be eliminated with evidence of tumor management or resolution, while press therapies could continue under modifications or adjustments (arrow). Optimization of timing, dosing, and scheduling of the press-pulse treatments will facilitate eradication of tumor cells with minimal patient toxicity. This therapeutic strategy is a framework for future clinical trials. HBOT, hyperbaric oxygen therapy; KD-R, calorie restricted ketogenic diet. The figure is reprinted with modifications, as described previously (34) and distributed under a Creative Commons license.

KMT has been used together with low dose chemotherapy and other treatments to manage tumor progression in a woman with stage IV triple negative breast cancer (154). The woman responded well to the combined treatment, and initially reported as a complete therapeutic response. Although overall survival exceeded the median expected for her stage and grade, she eventually succumbed to her cancer. A failure to continue with the KMT protocol was considered responsible in part for her tumor recurrence (154). Nevertheless, a subsequent clinical study showed that a ketogenic diet combined with carboplatin/paclitaxel, hyperthermia, and hyperbaric oxygen therapy significantly improved progression-free and overall survival in patients with advanced non-small cell lung cancer (211). Currently, neoadjuvant therapies are commonly prescribed for a subset of invasive or late-stage cancers, including breast cancers. KMT initiated soon after diagnosis and prior to surgery may prove to be a non-toxic adjunct treatment capable of downgrading the aggressive and invasive nature of the cancer, thereby increasing the efficacy of subsequent treatments (212). It is our view that improvements in selection, dosage, timing, and scheduling of drugs, diet, and procedures will offer benefits in survival and quality of life to patients with advanced metastatic breast cancer when used as a complementary or alternative therapeutic strategy alongside the SOC (Figure 5). The Press-Pulse therapeutic strategy for cancer management was based on the concept of Arens and West, who described how the simultaneous occurrence of “press-pulse” disturbances was responsible for the extinction of organic populations during prior evolutionary epochs (213). A similar concept can be used to show how tumors can be slowly degraded. Optimization of dosing, timing, and scheduling of KMT used together with synergistic drugs and procedures will facilitate the eradication of breast tumor cells with minimal patient toxicity. This therapeutic strategy can serve as a framework for the design of clinical trials for the non-toxic management of most cancers (34). Further details are described in Figure 5.

## Conclusions

Breast cancer, like many cancers, are dependent on fermentation metabolism for growth. Substrate level phosphorylation drives the fermentation metabolism of tumor cells using glucose and glutamine as major fuels in the cytoplasm and mitochondria, respectively. The protracted replacement of respiration with fermentation in cancer cells leads to unbridled cell proliferation. Fusion hybridization of epithelial-derived cancer stem cells with glutamine-dependent tissue macrophages is an alternative explanation to the epithelial-mesenchymal-transition for the origin of metastatic breast cancer cells. The aim of our review is to illustrate that the restriction of glucose and glutamine together with elevation of non-fermentable ketone bodies offers a complementary or alternative therapeutic strategy to the SOC for the non-toxic management of breast cancer. It is our view that KMT could improve progression-free and overall survival for most breast cancer patients.

## Author Contributions

TS conceived and wrote the review. MI, AS, MK, and J-PS contributed clinical information and edited the review. PM and CC contributed scientific information to the review.

### Conflict of Interest

MK was employed by Dietary Therapies LLC. The remaining authors declare that the research was conducted in the absence of any commercial or financial relationships that could be construed as a potential conflict of interest.

## Abbreviations

SLP: Substrate level phosphorylation
KMT: Ketogenic metabolic therapy
OxPhos: Oxidative phosphorylation
EMT: Epithelial mesenchymal transition
GKI: Glucose Ketone Index
SUCL: succinate-CoA ligase.

## References

[1] Siegel RL Miller KD Jemal A Cancer statistics, 2019. CA Cancer J Clin. (2019) 69:7–34. 10.3322/caac.21551 30620402

[2] Desantis CE Miller KD Goding Sauer A Jemal A Siegel RL. Cancer statistics for African Americans, 2019. CA Cancer J Clin. (2019) 69:211–33. 10.3322/caac.21555 30762872

[3] Greenman C Stephens P Smith R Dalgliesh GL Hunter C Bignell G. Patterns of somatic mutation in human cancer genomes. Nature. (2007) 446:153–8. 10.1038/nature05610 17344846 PMC2712719

[4] Hanahan D Weinberg RA. Hallmarks of cancer: the next generation. Cell. (2011) 144:646–74. 10.1016/j.cell.2011.02.013 21376230

[5] Vogelstein B Papadopoulos N Velculescu VE Zhou S Diaz LAJr Kinzler KW. Cancer genome landscapes. Science. (2013) 339:1546–58. 10.1126/science.1235122 23539594 PMC3749880

[6] Bayreuther K. Chromosomes in primary neoplastic growth. Nature. (1960) 186:6–9. 10.1038/186006a0 13797839

[7] Pitot HC Some biochemical aspects of malignancy. Ann Review Biochem. (1966) 35:335–68. 10.1146/annurev.bi.35.070166.002003

[8] Soto AM Sonnenschein C. The somatic mutation theory of cancer: growing problems with the paradigm? Bioessays. (2004) 26:1097–107. 10.1002/bies.20087 15382143

[9] Baker SG. A cancer theory kerfuffle can lead to new lines of research. J Natl Cancer Inst. (2015) 107:dju405. 10.1093/jnci/dju405 25528755 PMC4326310

[10] Parsons DW Jones S Zhang X Lin JC Leary RJ Angenendt P. An integrated genomic analysis of human glioblastoma multiforme. Science. (2008) 321:1807–12. 10.1126/science.1164382 18772396 PMC2820389

[11] Martincorena I Campbell PJ. Somatic mutation in cancer and normal cells. Science. (2015) 349:1483–9. 10.1126/science.aab4082 26404825

[12] Chanock SJ. The paradox of mutations and cancer. Science. (2018) 362:893–4. 10.1126/science.aav5697 30467157

[13] Martincorena I Fowler JC Wabik A Lawson ARJ Abascal F Hall MWJ. Somatic mutant clones colonize the human esophagus with age. Science. (2018) 362:911–7. 10.1126/science.aau3879 30337457 PMC6298579

[14] Yizhak K Aguet F Kim J Hess JM Kubler K Grimsby J. RNA sequence analysis reveals macroscopic somatic clonal expansion across normal tissues. Science. (2019) 364:eaaw0726. 10.1126/science.aaw0726 31171663 PMC7350423

[15] Yokoyama A Kakiuchi N Yoshizato T Nannya Y Suzuki H Takeuchi Y. Age-related remodelling of oesophageal epithelia by mutated cancer drivers. Nature. (2019) 565:312–7. 10.1038/s41586-018-0811-x 30602793

[16] Huttley GA Easteal S Southey MC Tesoriero A Giles GG Mccredie MR. Adaptive evolution of the tumour suppressor BRCA1 in humans and chimpanzees. Aus Breast Cancer Family Study Nat Genet. (2000) 25:410–3. 10.1038/78092 10932184

[17] Puente XS Velasco G Gutierrez-Fernandez A Bertranpetit J King MC Lopez-Otin C. Comparative analysis of cancer genes in the human and chimpanzee genomes. BMC Genomics. (2006) 7:15. 10.1186/1471-2164-7-15 16438707 PMC1382208

[18] Varki NM Varki A. On the apparent rarity of epithelial cancers in captive chimpanzees. Philos Trans R Soc Lond B Biol Sci. (2015) 370:20140225. 10.1098/rstb.2014.0225 26056369 PMC4581030

[19] Lowenstine LJ Mcmanamon R Terio KA. Comparative pathology of aging great apes: bonobos, chimpanzees, gorillas, and orangutans. Vet Pathol. (2016) 53:250–76. 10.1177/0300985815612154 26721908

[20] Kopp W. How western diet and lifestyle drive the pandemic of obesity and civilization diseases. Diabetes Metab Syndr Obes. (2019) 12:2221–36. 10.2147/DMSO.S216791 31695465 PMC6817492

[21] Seyfried TN. Cancer as a mitochondrial metabolic disease. Front Cell Dev Biol. (2015) 3:43. 10.3389/fcell.2015.00043 26217661 PMC4493566

[22] Kaipparettu BA Ma Y Park JH Lee TL Zhang Y Yotnda P. Crosstalk from non-cancerous mitochondria can inhibit tumor properties of metastatic cells by suppressing oncogenic pathways. PLoS ONE. (2013) 8:e61747. 10.1371/journal.pone.0061747 23671572 PMC3650012

[23] Chang JC Chang HS Wu YC Cheng WL Lin TT Chang HJ. Mitochondrial transplantation regulates antitumour activity, chemoresistance and mitochondrial dynamics in breast cancer. J Exp Clin Cancer Res. (2019) 38:30. 10.1186/s13046-019-1028-z 30674338 PMC6343292

[24] Isidoro A Casado E Redondo A Acebo P Espinosa E Alonso AM. Breast carcinomas fulfill the Warburg hypothesis and provide metabolic markers of cancer prognosis. Carcinogenesis. (2005) 26:2095–104. 10.1093/carcin/bgi188 16033770

[25] Petros JA Baumann AK Ruiz-Pesini E Amin MB Sun CQ Hall J. mtDNA mutations increase tumorigenicity in prostate cancer. Proc Natl Acad Sci USA. (2005) 102:719–24. 10.1073/pnas.0408894102 15647368 PMC545582

[26] Ayyasamy V Owens KM Desouki MM Liang P Bakin A Thangaraj K. Cellular model of warburg effect identifies tumor promoting function of UCP2 in breast cancer and its suppression by genipin. PLoS ONE. (2011) 6:e24792. 10.1371/journal.pone.0024792 21935467 PMC3174207

[27] Owens KM Kulawiec M Desouki MM Vanniarajan A Singh KK. Impaired OXPHOS complex III in breast cancer. PLoS ONE. (2011) 6:e23846. 10.1371/journal.pone.0023846 21901141 PMC3162009

[28] Hu Y Lu W Chen G Wang P Chen Z Zhou Y. K-ras(G12V) transformation leads to mitochondrial dysfunction and a metabolic switch from oxidative phosphorylation to glycolysis. Cell Res. (2012) 22:399–412. 10.1038/cr.2011.145 21876558 PMC3257361

[29] Putignani L Raffa S Pescosolido R Rizza T Del Chierico F Leone L. Preliminary evidences on mitochondrial injury and impaired oxidative metabolism in breast cancer. Mitochondrion. (2012) 12:363–9. 10.1016/j.mito.2012.02.003 22366096

[30] Seyfried TN Cancer as a Metabolic Disease: On the Origin, Management, and Prevention of Cancer. Hoboken, NJ: John Wiley & Sons (2012).

[31] Verschoor ML Ungard R Harbottle A Jakupciak JP Parr RL Singh G. Mitochondria and cancer: past, present, and future. Biomed Res Int. (2013) 2013:612369. 10.1155/2013/612369 23509753 PMC3581248

[32] Seyfried TN Flores RE Poff AM D'agostino DP. Cancer as a metabolic disease: implications for novel therapeutics. Carcinogenesis. (2014) 35:515–27. 10.1093/carcin/bgt480 24343361 PMC3941741

[33] Srinivasan S Guha M Dong DW Whelan KA Ruthel G Uchikado Y. Disruption of cytochrome c oxidase function induces the warburg effect and metabolic reprogramming. Oncogene. (2016) 35:1585–95. 10.1038/onc.2015.227 26148236 PMC4703574

[34] Seyfried TN Yu G Maroon JC D'agostino DP. Press-pulse: a novel therapeutic strategy for the metabolic management of cancer. Nutr Metab. (2017) 14:19. 10.1186/s12986-017-0178-2 28250801 PMC5324220

[35] Da Silva I Da Costa Vieira R Stella C Loturco E Carvalho AL Veo C. Inborn-like errors of metabolism are determinants of breast cancer risk, clinical response and survival: a study of human biochemical individuality. Oncotarget. (2018) 9:31664–81. 10.18632/oncotarget.25839 30167086 PMC6114970

[36] Rouiller C. Physiological and pathological changes in mitochondrial morphology. Int Rev Cytol. (1960) 9:227–92. 10.1016/S0074-7696(08)62748-5 14439544

[37] Roddy HJ Silverberg SG. Ultrastructural analysis of apocrine carcinoma of the human breast. Ultrastruct Pathol. (1980) 1:385–93. 10.3109/01913128009141441 7233593

[38] Gadaleanu V Craciun C. Malignant oncocytoma of the breast. Zentralbl Allg Pathol. (1987) 133:279–83. 3630436

[39] Pelicano H Xu RH Du M Feng L Sasaki R Carew JS. Mitochondrial respiration defects in cancer cells cause activation of Akt survival pathway through a redox-mediated mechanism. J Cell Biol. (2006) 175:913–23. 10.1083/jcb.200512100 17158952 PMC2064701

[40] Elliott RL Jiang XP Head JF. Mitochondria organelle transplantation: introduction of normal epithelial mitochondria into human cancer cells inhibits proliferation and increases drug sensitivity. Breast Cancer Res Treat. (2012) 136:347–54. 10.1007/s10549-012-2283-2 23080556

[41] Zeczycki TN Whelan J Hayden WT Brown DA Shaikh SR. Increasing levels of cardiolipin differentially influence packing of phospholipids found in the mitochondrial inner membrane. Biochem Biophys Res Commun. (2014) 450:366–71. 10.1016/j.bbrc.2014.05.133 24905496

[42] Jogalekar MP Serrano EE. Morphometric analysis of a triple negative breast cancer cell line in hydrogel and monolayer culture environments. PeerJ. (2018) 6:e4340. 10.7717/peerj.4340 29473000 PMC5817938

[43] Putignani L Raffa S Pescosolido R Aimati L Signore F Torrisi MR. Alteration of expression levels of the oxidative phosphorylation system. (OXPHOS) in breast cancer cell mitochondria. Breast Cancer Res Treat. (2008) 110:439–52. 10.1007/s10549-007-9738-x 17899367

[44] Arismendi-Morillo G Castellano-Ramirez A Seyfried TN. Ultrastructural characterization of the Mitochondria-associated membranes abnormalities in human astrocytomas: functional and therapeutics implications. Ultrastruct Pathol. (2017) 41:234–44. 10.1080/01913123.2017.1300618 28375672

[45] Morciano G Marchi S Morganti C Sbano L Bittremieux M Kerkhofs M. Role of mitochondria-associated ER membranes in calcium regulation in cancer-specific settings. Neoplasia. (2018) 20:510–23. 10.1016/j.neo.2018.03.005 29626751 PMC5916088

[46] Chinopoulos C Seyfried TN. Mitochondrial substrate level phosphorylation as energy source for glioblastoma: review and hypothesis. ASN Neuro. (2018) 10:1–27. 10.1177/1759091418818261 30909720 PMC6311572

[47] Warburg O Wind F Negelein E. The metabolism of tumors in the body. J Gen Physiol. (1927) 8:519–30. 10.1085/jgp.8.6.519 19872213 PMC2140820

[48] Warburg O The Metabolism of Tumours. New York, NY: Richard R Smith (1931).

[49] Warburg O. On the origin of cancer cells. Science. (1956) 123:309–14. 10.1126/science.123.3191.309 13298683

[50] Warburg O. On the respiratory impairment in cancer cells. Science. (1956) 124:269–70. 13351639

[51] Warburg O Revidsed lindau lectures: the prime cause of cancer and prevention - parts 1 & 2, In: Burk D, editor. Meeting of the Nobel-Laureates. Lindau: K.Triltsch (1969). p. 1–9.

[52] Racker E. Bioenergetics and the problem of tumor growth. Am Sci. (1972) 60:56–63. 4332766

[53] Selak MA Armour SM Mackenzie ED Boulahbel H Watson DG Mansfield KD. Succinate links TCA cycle dysfunction to oncogenesis by inhibiting HIF-alpha prolyl hydroxylase. Cancer Cell. (2005) 7:77–85. 10.1016/j.ccr.2004.11.022 15652751

[54] Tannahill GM Curtis AM Adamik J Palsson-Mcdermott EM Mcgettrick AF Goel G. Succinate is an inflammatory signal that induces IL-1beta through HIF-1alpha. Nature. (2013) 496:238–42. 10.1038/nature11986 23535595 PMC4031686

[55] Chouchani ET Pell VR Gaude E Aksentijevic D Sundier SY Robb EL. Ischaemic accumulation of succinate controls reperfusion injury through mitochondrial ROS. Nature. (2014) 515:431–5. 10.1038/nature13909 25383517 PMC4255242

[56] Semenza GL. Hypoxia-inducible factors: coupling glucose metabolism and redox regulation with induction of the breast cancer stem cell phenotype. EMBO J. (2017) 36:252–9. 10.15252/embj.201695204 28007895 PMC5286373

[57] Gao C Shen Y Jin F Miao Y Qiu X. Cancer stem cells in small cell lung cancer cell line H446: higher dependency on oxidative phosphorylation and mitochondrial substrate-level phosphorylation than non-stem cancer cells. PLoS ONE. (2016) 11:e0154576. 10.1371/journal.pone.0154576 27167619 PMC4863974

[58] Chen Q Kirk K Shurubor YI Zhao D Arreguin AJ Shahi I. Rewiring of glutamine metabolism is a bioenergetic adaptation of human cells with mitochondrial DNA mutations. Cell Metab. (2018) 27:1007–25 e1005. 10.1016/j.cmet.2018.03.002 29657030 PMC5932217

[59] Flores RE Brown AK Taus L Khoury J Glover F Kami K. Mycoplasma infection and hypoxia initiate succinate accumulation and release in the VM-M3 cancer cells. Biochim Biophys Acta. (2018) 1859:975–83. 10.1016/j.bbabio.2018.03.012 29580805

[60] Mukherjee P Augur ZM Li M Hill C Greenwood B Domin MA. Therapeutic benefit of combining calorie-restricted ketogenic diet and glutamine targeting in late-stage experimental glioblastoma. Commun Biol. (2019) 2:200. 10.1038/s42003-019-0455-x 31149644 PMC6541653

[61] Vander Heiden MG Locasale JW Swanson KD Sharfi H Heffron GJ Amador-Noguez D. Evidence for an alternative glycolytic pathway in rapidly proliferating cells. Science. (2010) 329:1492–9. 10.1126/science.1188015 20847263 PMC3030121

[62] Israelsen WJ Dayton TL Davidson SM Fiske BP Hosios AM Bellinger G. PKM2 isoform-specific deletion reveals a differential requirement for pyruvate kinase in tumor cells. Cell. (2013) 155:397–409. 10.1016/j.cell.2013.09.025 24120138 PMC3850755

[63] Yu M Chen S Hong W Gu Y Huang B Lin Y. Prognostic role of glycolysis for cancer outcome: evidence from 86 studies. J Cancer Res Clin Oncol. (2019) 145:967–99. 10.1007/s00432-019-02847-w 30825027 PMC11810312

[64] Seyfried TN Shelton LM. Cancer as a metabolic disease. Nutr Metab. (2010) 7:7. 10.1186/1743-7075-7-7 20181022 PMC2845135

[65] Seyfried TN Is mitochondrial glutamine fermentation a missing link in the metabolic theory of cancer? In: Cancer as a Metabolic Disease: On the Origin, Management, and Prevention of Cancer. Hoboken, NJ: John Wiley & Sons (2012). p. 133–144. 10.1002/9781118310311.ch8

[66] Merchant TE Gierke LW Meneses P Glonek T. 31P magnetic resonance spectroscopic profiles of neoplastic human breast tissues. Cancer Res. (1988) 48:5112–8. 3409237

[67] Semenza GL Artemov D Bedi A Bhujwalla Z Chiles K Feldser D. The metabolism of tumours: 70 years later. Novartis Found Symp. (2001) 240:251–60. 10.1002/0470868716.ch17 11727934

[68] Isidoro A Martinez M Fernandez PL Ortega AD Santamaria G Chamorro M. Alteration of the bioenergetic phenotype of mitochondria is a hallmark of breast, gastric, lung and oesophageal cancer. Biochem J. (2004) 378:17–20. 10.1042/bj20031541 14683524 PMC1223948

[69] Robey IF Stephen RM Brown KS Baggett BK Gatenby RA Gillies RJ. Regulation of the warburg effect in early-passage breast cancer cells. Neoplasia. (2008) 10:745–56. 10.1593/neo.07724 18670636 PMC2481565

[70] Palaskas N Larson SM Schultz N Komisopoulou E Wong J Rohle D. 18F-fluorodeoxy-glucose positron emission tomography marks MYC-overexpressing human basal-like breast cancers. Cancer Res. (2011) 71:5164–74. 10.1158/0008-5472.CAN-10-4633 21646475 PMC3148325

[71] Pelicano H Zhang W Liu J Hammoudi N Dai J Xu RH. Mitochondrial dysfunction in some triple-negative breast cancer cell lines: role of mTOR pathway and therapeutic potential. Breast Cancer Res. (2014) 16:434. 10.1186/s13058-014-0434-6 25209360 PMC4303115

[72] Madonna MC Fox DB Crouch BT Lee J Zhu C Martinez AF. Optical imaging of glucose uptake and mitochondrial membrane potential to characterize Her2 breast tumor metabolic phenotypes. Mol Cancer Res. (2019) 17:1545–55. 10.1158/1541-7786.MCR-18-0618 30902832 PMC6610750

[73] Reis LMD Adamoski D Ornitz Oliveira Souza R Rodrigues Ascencao CF Sousa De Oliveira KR Correa-Da-Silva F. Dual inhibition of glutaminase and carnitine palmitoyltransferase decreases growth and migration of glutaminase inhibition-resistant triple-negative breast cancer cells. J Biol Chem. (2019) 294:9342–57. 10.1074/jbc.RA119.008180 31040181 PMC6579458

[74] Rosso M Lapyckyj L Besso MJ Monge M Reventos J Canals F. Characterization of the molecular changes associated with the overexpression of a novel epithelial cadherin splice variant mRNA in a breast cancer model using proteomics and bioinformatics approaches: identification of changes in cell metabolism and an increased expression of lactate dehydrogenase B. Cancer Metab. (2019) 7:5. 10.1186/s40170-019-0196-9 31086659 PMC6507066

[75] Katt WP Ramachandran S Erickson JW Cerione RA. Dibenzophenanthridines as inhibitors of glutaminase C and cancer cell proliferation. Mol Cancer Ther. (2012) 11:1269–78. 10.1158/1535-7163.MCT-11-0942 22496480 PMC3620022

[76] Van Geldermalsen M Wang Q Nagarajah R Marshall AD Thoeng A Gao D. ASCT2/SLC1A5 controls glutamine uptake and tumour growth in triple-negative basal-like breast cancer. Oncogene. (2016) 35:3201–8. 10.1038/onc.2015.381 26455325 PMC4914826

[77] Demas DM Demo S Fallah Y Clarke R Nephew KP Althouse S. Glutamine metabolism drives growth in advanced hormone receptor positive breast cancer. Front Oncol. (2019) 9:686. 10.3389/fonc.2019.00686 31428575 PMC6688514

[78] Gwangwa MV Joubert AM Visagie MH. Effects of glutamine deprivation on oxidative stress and cell survival in breast cell lines. Biol Res. (2019) 52:15. 10.1186/s40659-019-0224-9 30917872 PMC6437944

[79] Mayr E The Growth of Biological Thought: Diversity, Evolution, and Inheritance. Cambridge: Belknap Harvard (1982).

[80] Schlesinger AB Explaining Life. New York, NY: McGraw-Hill, Inc (1994).

[81] Seyfried TN Nothing in cancer biology makes sense except in the light of evolution In: *Cancer as a Metabolic Disease: On the Origin, Management, and Prevention of Cancer* Hoboken, NJ: John Wiley & Sons (2012). p. 261–75. 10.1002/9781118310311.ch15

[82] Sonnenschein C Soto AM. Somatic mutation theory of carcinogenesis: why it should be dropped and replaced. Mol Carcinog. (2000) 29:205–211. 10.1002/1098-2744(200012)29:4>205::AID-MC1002<3.0.CO;2-W 11170258

[83] Szent-Gyorgyi A. The living state and cancer. Proc Natl Acad Sci USA. (1977) 74:2844–7. 10.1073/pnas.74.7.2844 268635 PMC431314

[84] Seyfried TN Shelton L Arismendi-Morillo G Kalamian M Elsakka A Maroon J. Provocative question: should ketogenic metabolic therapy become the standard of care for glioblastoma? Neurochem Res. (2019) 44:2392–404. 10.1007/s11064-019-02795-4 31025151

[85] Klaunig JE Kamendulis LM Hocevar BA. Oxidative stress and oxidative damage in carcinogenesis. Toxicol Pathol. (2010) 38:96–109. 10.1177/0192623309356453 20019356

[86] Iglesias P Salas A Costoya JA. The maintenance of mitochondrial genetic stability is crucial during the oncogenic process. Commun Integ Biol. (2012) 5:34–8. 10.4161/cib.18160 22482007 PMC3291311

[87] Galadari S Rahman A Pallichankandy S Thayyullathil F Reactive oxygen species and cancer paradox: to promote or to suppress? Free Radic Biol Med. (2017) 104:144–64. 10.1016/j.freeradbiomed.2017.01.004 28088622

[88] Desler C Lykke A Rasmussen LJ. The effect of mitochondrial dysfunction on cytosolic nucleotide metabolism. J Nucleic Acids. (2010) 2010:701518. 10.4061/2010/701518 20862377 PMC2938461

[89] Ralph SJ Rodriguez-Enriquez S Neuzil J Saavedra E Moreno-Sanchez R. The causes of cancer revisited: mitochondrial malignancy and ROS-induced oncogenic transformation - why mitochondria are targets for cancer therapy. Mol Aspects Med. (2010) 31:145–70. 10.1016/j.mam.2010.02.008 20206201

[90] Valle A Oliver J Roca P. Role of uncoupling proteins in cancer. Cancers. (2010) 2:567–91. 10.3390/cancers2020567 24281083 PMC3835092

[91] Degtyareva NP Heyburn L Sterling J Resnick MA Gordenin DA Doetsch PW. Oxidative stress-induced mutagenesis in single-strand DNA occurs primarily at cytosines and is DNA polymerase zeta-dependent only for adenines and guanines. Nucleic Acids Res. (2013) 41:8995–9005. 10.1093/nar/gkt671 23925127 PMC3799438

[92] Bartesaghi S Graziano V Galavotti S Henriquez NV Betts J Saxena J. Inhibition of oxidative metabolism leads to p53 genetic inactivation and transformation in neural stem cells. Proc Natl Acad Sci USA. (2015) 112:1059–64. 10.1073/pnas.1413165112 25583481 PMC4313844

[93] Rodic S Vincent MD. Reactive oxygen species. (ROS) are a key determinant of cancer's metabolic phenotype. Int J Cancer. (2018) 142:440–8. 10.1002/ijc.31069 28940517

[94] Lu H Forbes RA Verma A. Hypoxia-inducible factor 1 activation by aerobic glycolysis implicates the warburg effect in carcinogenesis. J Biol Chem. (2002) 277:23111–5. 10.1074/jbc.M202487200 11943784

[95] Matoba S Kang JG Patino WD Wragg A Boehm M Gavrilova O. p53 regulates mitochondrial respiration. Science. (2006) 312:1650–3. 10.1126/science.1126863 16728594

[96] Lu W Pelicano H Huang P. Cancer metabolism: is glutamine sweeter than glucose? Cancer Cell. (2010) 18:199–200. 10.1016/j.ccr.2010.08.017 20832746 PMC2952340

[97] Yang D Wang MT Tang Y Chen Y Jiang H Jones TT Impairment of mitochondrial respiration in mouse fibroblasts by oncogenic H-RAS^Q61L^. Cancer Biol Ther. (2010) 9:122–33. 10.4161/cbt.9.2.10379 19923925 PMC2909490

[98] Nicolay BN Danielian PS Kottakis F Lapek JD Jr Sanidas I Miles WO. Proteomic analysis of pRb loss highlights a signature of decreased mitochondrial oxidative phosphorylation. Genes Dev. (2015) 29:1875–89. 10.1101/gad.264127.115 26314710 PMC4573859

[99] Szeliga M Albrecht J. Opposing roles of glutaminase isoforms in determining glioblastoma cell phenotype. Neurochem Int. (2015) 88:6–9. 10.1016/j.neuint.2014.11.004 25529918

[100] Seyfried TN Energetics of normal cells and cancer cells In: Cancer as a Metabolic Disease: On the Origin, Management, and Prevention of Cancer. Hoboken, NJ: John Wiley & Sons (2012). p. 47–72. 10.1002/9781118310311.ch4

[101] Zhang C Liu J Liang Y Wu R Zhao Y Hong X. Tumour-associated mutant p53 drives the warburg effect. Nat Commun. (2013) 4:2935. 10.1038/ncomms3935 24343302 PMC3969270

[102] Reynolds MR Lane AN Robertson B Kemp S Liu Y Hill BG. Control of glutamine metabolism by the tumor suppressor Rb. Oncogene. (2014) 33:556–66. 10.1038/onc.2012.635 23353822 PMC3918885

[103] Osborne C Wilson P Tripathy D. Oncogenes and tumor suppressor genes in breast cancer: potential diagnostic and therapeutic applications. Oncologist. (2004) 9:361–77. 10.1634/theoncologist.9-4-361 15266090

[104] Pereira B Chin SF Rueda OM Vollan HK Provenzano E Bardwell HA. The somatic mutation profiles of 2,433 breast cancers refines their genomic and transcriptomic landscapes. Nat Commun. (2016) 7:11479. 10.1038/ncomms11479 27161491 PMC4866047

[105] Zhang D Tai LK Wong LL Chiu LL Sethi SK Koay ES. Proteomic study reveals that proteins involved in metabolic and detoxification pathways are highly expressed in HER-2/neu-positive breast cancer. Mol Cell Proteomics. (2005) 4:1686–96. 10.1074/mcp.M400221-MCP200 16048908

[106] Ding Y Liu Z Desai S Zhao Y Liu H Pannell LK. Receptor tyrosine kinase ErbB2 translocates into mitochondria and regulates cellular metabolism. Nat Commun. (2012) 3:1271. 10.1038/ncomms2236 23232401 PMC3521558

[107] Privat M Radosevic-Robin N Aubel C Cayre A Penault-Llorca F Marceau G. BRCA1 induces major energetic metabolism reprogramming in breast cancer cells. PLoS ONE. (2014) 9:e102438. 10.1371/journal.pone.0102438 25010005 PMC4092140

[108] Cuyas E Fernandez-Arroyo S Alarcon T Lupu R Joven J Menendez JA. Germline BRCA1 mutation reprograms breast epithelial cell metabolism towards mitochondrial-dependent biosynthesis: evidence for metformin-based starvation strategies in BRCA1 carriers. Oncotarget. (2016) 7:52974–92. 10.18632/oncotarget.9732 27259235 PMC5288162

[109] Seyfried TN Huysentruyt LC. On the origin of cancer metastasis. Crit Rev Oncogene. (2013) 18:43–73. 10.1615/CritRevOncog.v18.i1-2.40 23237552 PMC3597235

[110] Lazebnik Y. What are the hallmarks of cancer? Nat Rev Cancer. (2010) 10:232–3. 10.1038/nrc2827 20355252

[111] Fidler IJ. The pathogenesis of cancer metastasis: the seed and soil hypothesis revisited. Nat Rev Cancer. (2003) 3:453–8. 10.1038/nrc1098 12778135

[112] Weinberg RA The Biology of Cancer. New York, NY: Garland Science (2007).

[113] Kar R Jha NK Jha SK Sharma A Dholpuria S Asthana N. A NOTCH Deeper into the epithelial-to-mesenchymal transition. (EMT) program in breast cancer. Genes. (2019) 10:961. 10.3390/genes10120961 31766724 PMC6947643

[114] Pawelek JM. Cancer-cell fusion with migratory bone-marrow-derived cells as an explanation for metastasis: new therapeutic paradigms. Fut Oncol. (2008) 4:449–52. 10.2217/14796694.4.4.449 18684055

[115] Pawelek JM Chakraborty AK. The cancer cell–leukocyte fusion theory of metastasis. Adv Cancer Res. (2008) 101:397–444. 10.1016/S0065-230X(08)00410-7 19055949

[116] Powell AE Anderson EC Davies PS Silk AD Pelz C Impey S. Fusion between Intestinal epithelial cells and macrophages in a cancer context results in nuclear reprogramming. Cancer Res. (2011) 71:1497–505. 10.1158/0008-5472.CAN-10-3223 21303980 PMC3079548

[117] Gast CE Silk AD Zarour L Riegler L Burkhart JG Gustafson KT. Cell fusion potentiates tumor heterogeneity and reveals circulating hybrid cells that correlate with stage and survival. Sci Adv. (2018) 4:eaat7828. 10.1126/sciadv.aat7828 30214939 PMC6135550

[118] Marin-Padilla M. Erythrophagocytosis by epithelial cells of a breast carcinoma. Cancer. (1977) 39:1085–1089. 10.1002/1097-0142(197703)39:3>1085::AID-CNCR2820390312<3.0.CO;2-U 199342

[119] Ruff MR Pert CB. Small cell carcinoma of the lung: macrophage-specific antigens suggest hemopoietic stem cell origin. Science. (1984) 225:1034–6. 10.1126/science.6089338 6089338

[120] Ruff MR Pert CB. Origin of human small cell lung cancer. Science. (1985) 229:680. 10.1126/science.229.4714.680 17739381

[121] Calvo F Martin PM Jabrane N De Cremoux P Magdelenat H. Human breast cancer cells share antigens with the myeloid monocyte lineage. Br J Cancer. (1987) 56:15–9. 10.1038/bjc.1987.145 3304388 PMC2001668

[122] Kobayashi M Sugimoto T Okabayashi T Okamoto K Namikawa T Tochika N. Localization of thymidine phosphorylase in breast cancer tissue. Med Mol Morphol. (2005) 38:112–7. 10.1007/s00795-005-0282-7 15944818

[123] Huysentruyt LC Seyfried TN. Perspectives on the mesenchymal origin of metastatic cancer. Cancer Metastasis Rev. (2010) 29:695–707. 10.1007/s10555-010-9254-z 20839033 PMC2962789

[124] Schramm HM. Should EMT of cancer cells be understood as epithelial-myeloid transition? J Cancer. (2014) 5:125–32. 10.7150/jca.8242 24494030 PMC3909767

[125] Rowan DJ Logunova V Van Tuinen P Olteanu H Peterson JF. Circulating breast carcinoma cells mimicking therapy-related acute myeloid leukemia. Int J Surg Pathol. (2017) 25:87–93. 10.1177/1066896916664986 27543510

[126] Wu JM Fackler MJ Halushka MK Molavi DW Taylor ME Teo WW. Heterogeneity of breast cancer metastases: comparison of therapeutic target expression and promoter methylation between primary tumors and their multifocal metastases. Clin Cancer Res. (2008) 14:1938–46. 10.1158/1078-0432.CCR-07-4082 18381931 PMC2965068

[127] Weigelt B Reis-Filho JS. Histological and molecular types of breast cancer: is there a unifying taxonomy? Nat Rev Clin Oncol. (2009) 6:718–30. 10.1038/nrclinonc.2009.166 19942925

[128] Paget S The distribution of secondary growths in cancer of the breast. Lancet. (1889) 1:571–3. 10.1016/S0140-6736(00)49915-0 2673568

[129] Kawaguchi T. Organ preference of cancer metastasis and metastasis-related cell adhesion molecules including carbohydrates. Cardiovasc Hematol Disord Drug Targets. (2016) 15:164–86. 10.2174/1871529X15666151102102551 26521885

[130] Newsholme P Why is L-glutamine metabolism important to cells of the immune system in health, postinjury, surgery or infection? J Nutr. (2001) 131:2515S−22S. 10.1093/jn/131.9.2515S 11533304

[131] Ren W Xia Y Chen S Wu G Bazer FW Zhou B. Glutamine metabolism in macrophages: a novel target for obesity/type 2 diabetes. Adv Nutr. (2019) 10:321–30. 10.1093/advances/nmy084 30753258 PMC6416106

[132] Wallis M Tardivon A Helbich T Schreer I European Society of Breast I. Guidelines from the European society of breast imaging for diagnostic interventional breast procedures. Eur Radiol. (2007) 17:581–8. 10.1007/s00330-006-0408-x 17013595

[133] Kurian AW Lichtensztajn DY Keegan TH Nelson DO Clarke CA Gomez SL. Use of and mortality after bilateral mastectomy compared with other surgical treatments for breast cancer in California, 1998-2011. JAMA. (2014) 312:902–14. 10.1001/jama.2014.10707 25182099 PMC5747359

[134] Hofer SO Molema G Hermens RA Wanebo HJ Reichner JS Hoekstra HJ. The effect of surgical wounding on tumour development. Eur J Surg Oncol. (1999) 25:231–43. 10.1053/ejso.1998.0634 10336800

[135] Tagliabue E Agresti R Carcangiu ML Ghirelli C Morelli D Campiglio M. Role of HER2 in wound-induced breast carcinoma proliferation. Lancet. (2003) 362:527–33. 10.1016/S0140-6736(03)14112-8 12932384

[136] Baum M. Does the act of surgery provoke activation of latent metastases in early breast cancer? Breast Cancer Res. (2004) 6:160–1. 10.1186/bcr902 15217487 PMC468671

[137] Demicheli R Retsky MW Hrushesky WJ Baum M Gukas ID. The effects of surgery on tumor growth: a century of investigations. Ann Oncol. (2008) 19:1821–8. 10.1093/annonc/mdn386 18550576

[138] Walter ND Rice PL Redente EF Kauvar EF Lemond L Aly T. Wound healing after trauma may predispose to lung cancer metastasis: review of potential mechanisms. Am J Respir Cell Mol Biol. (2011) 44:591–6. 10.1165/rcmb.2010-0187RT 21177982

[139] Hobson J Gummadidala P Silverstrim B Grier D Bunn J James T. Acute inflammation induced by the biopsy of mouse mammary tumors promotes the development of metastasis. Breast Cancer Res Treat. (2013) 139:391–401. 10.1007/s10549-013-2575-1 23715631 PMC4038002

[140] Szalayova G Ogrodnik A Spencer B Wade J Bunn J Ambaye A. Human breast cancer biopsies induce eosinophil recruitment and enhance adjacent cancer cell proliferation. Breast Cancer Res Treat. (2016) 157:461–74. 10.1007/s10549-016-3839-3 27249999 PMC5026505

[141] Singer CF Hudelist G Fuchs EM Kostler W Fink-Retter A Gschwantler-Kaulich D. Incomplete surgical resection of ductal carcinomas *in situ* results in activation of ERBB2 in residual breast cancer cells. Endocr Relat Cancer. (2009) 16:73–83. 10.1677/ERC-08-0065 18948375

[142] Muti P Quattrin T Grant BJ Krogh V Micheli A Schunemann HJ. Fasting glucose is a risk factor for breast cancer: a prospective study. Cancer Epidemiol Biomarkers Prev. (2002) 11:1361–8. 12433712

[143] Krone CA Ely JT. Controlling hyperglycemia as an adjunct to cancer therapy. Integr Cancer Ther. (2005) 4:25–31. 10.1177/1534735404274167 15695475

[144] Rapp K Schroeder J Klenk J Ulmer H Concin H Diem G. Fasting blood glucose and cancer risk in a cohort of more than 140,000 adults in Austria. Diabetologia. (2006) 49:945–52. 10.1007/s00125-006-0207-6 16557372

[145] Alokail MS Al-Daghri N Abdulkareem A Draz HM Yakout SM Alnaami AM. Metabolic syndrome biomarkers and early breast cancer in Saudi women: evidence for the presence of a systemic stress response and/or a pre-existing metabolic syndrome-related neoplasia risk? BMC Cancer. (2013) 13:54. 10.1186/1471-2407-13-54 23374911 PMC3571930

[146] Lende TH Austdal M Bathen TF Varhaugvik AE Skaland I Gudlaugsson E. Metabolic consequences of perioperative oral carbohydrates in breast cancer patients - an explorative study. BMC Cancer. (2019) 19:1183. 10.1186/s12885-019-6393-7 31801490 PMC6894229

[147] Li W Zhang X Sang H Zhou Y Shang C Wang Y. Effects of hyperglycemia on the progression of tumor diseases. J Exp Clin Cancer Res. (2019) 38:327. 10.1186/s13046-019-1309-6 31337431 PMC6651927

[148] Windelov JA Pedersen J Holst JJ. Use of anesthesia dramatically alters the oral glucose tolerance and insulin secretion in C57Bl/6 mice. Physiol Rep. (2016) 4:e12824. 10.14814/phy2.12824 27255361 PMC4908499

[149] Guo NL Zhang JX Wu JP Xu YH. Isoflurane promotes glucose metabolism through up-regulation of miR-21 and suppresses mitochondrial oxidative phosphorylation in ovarian cancer cells. Biosci Rep. (2017) 37:BSR20170818. 10.1042/BSR20170818 28951521 PMC5725613

[150] Fan P Siwak DR Abderrahman B Agboke FA Yerrum S Jordan VC. Suppression of nuclear factor- κb by glucocorticoid receptor blocks estrogen-induced apoptosis in estrogen-deprived breast cancer cells. Mol Cancer Ther. (2019) 18:1684–95. 10.1158/1535-7163.MCT-18-1363 31511352 PMC6774891

[151] Gluschnaider U Hertz R Ohayon S Smeir E Smets M Pikarsky E. Long-chain fatty acid analogues suppress breast tumorigenesis and progression. Cancer Res. (2014) 74:6991–7002. 10.1158/0008-5472.CAN-14-0385 25304261

[152] Branca JJ Pacini S Ruggiero M. Effects of pre-surgical vitamin d supplementation and ketogenic diet in a patient with recurrent breast cancer. Anticancer Res. (2015) 35:5525–32. 26408720

[153] Hyde PN Lustberg MB Miller VJ Lafountain RA Volek JS Pleiotropic effects of nutritional ketosis: conceptual framework for ketoadaptation as a breast cancer therapy. Cancer Treat Res Comm. (2017) 12:32–9. 10.1016/j.ctarc.2017.06.001

[154] Iyikesici MS Slocum AK Slocum A Berkarda FB Kalamian M Seyfried TN. Efficacy of metabolically supported chemotherapy combined with ketogenic diet, hyperthermia, and hyperbaric oxygen therapy for stage IV triple-negative breast cancer. Cureus. (2017) 9:e1445. 10.7759/cureus.1445 28924531 PMC5589510

[155] Klement RJ Bandyopadhyay PS Champ CE Walach H. Application of Bayesian evidence synthesis to modelling the effect of ketogenic therapy on survival of high grade glioma patients. Theor Biol Med Model. (2018) 15:12. 10.1186/s12976-018-0084-y 30122157 PMC6100754

[156] Cohen CW Fontaine KR Arend RC Gower BA A ketogenic diet is acceptable in women with ovarian and endometrial cancer and has no adverse effects on blood lipids: a randomized, controlled trial. Nutr Cancer. (2019) 27:1–11. 10.1080/01635581.2019.1645864 31352797

[157] Khodabakhshi A Akbari ME Mirzaei HR Mehrad-Majd H Kalamian M Davoodi SH Feasibility, safety, and beneficial effects of MCT-based ketogenic diet for breast cancer treatment: a randomized controlled trial study. Nutr Cancer. (2019) 9:1–8. 10.1080/01635581.2019.1650942 31496287

[158] Licha D Vidali S Aminzadeh-Gohari S Alka O Breitkreuz L Kohlbacher O. Untargeted metabolomics reveals molecular effects of ketogenic diet on healthy and tumor xenograft mouse models. Int J Mol Sci. (2019) 20:3873. 10.3390/ijms20163873 31398922 PMC6719192

[159] Mitchell T Clarke L Goldberg A Bishop KS. Pancreatic cancer cachexia: the role of nutritional interventions. Healthcare. (2019) 7:89. 10.3390/healthcare7030089 31323984 PMC6787643

[160] Weber DD Aminzadeh-Gohari S Tulipan J Catalano L Feichtinger RG Kofler B Ketogenic diet in the treatment of cancer - Where do we stand? Mol Metab. (2019) 118:668–88. 10.1016/j.molmet.2019.06.026 PMC7056920 31399389

[161] Bartmann C Janaki Raman SR Floter J Schulze A Bahlke K Willingstorfer J Beta-hydroxybutyrate. (3-OHB) can influence the energetic phenotype of breast cancer cells, but does not impact their proliferation and the response to chemotherapy or radiation. Cancer Metab. (2018) 6:8 10.1186/s40170-018-0180-9 29942509 PMC5996481

[162] Bonuccelli G Tsirigos A Whitaker-Menezes D Pavlides S Pestell RG Chiavarina B. Ketones and lactate “fuel” tumor growth and metastasis: evidence that epithelial cancer cells use oxidative mitochondrial metabolism. Cell Cycle. (2010) 9:3506–14. 10.4161/cc.9.17.12731 20818174 PMC3047616

[163] Zheng J. Energy metabolism of cancer: glycolysis versus oxidative phosphorylation. Oncol Lett. (2012) 4:1151–7. 10.3892/ol.2012.928 23226794 PMC3506713

[164] Pascual G Avgustinova A Mejetta S Martin M Castellanos A Attolini CS. Targeting metastasis-initiating cells through the fatty acid receptor CD36. Nature. (2017) 541:41–5. 10.1038/nature20791 27974793

[165] Rodrigues LM Uribe-Lewis S Madhu B Honess DJ Stubbs M Griffiths JR. The action of beta-hydroxybutyrate on the growth, metabolism and global histone H3 acetylation of spontaneous mouse mammary tumours: evidence of a beta-hydroxybutyrate paradox. Cancer Metab. (2017) 5:4. 10.1186/s40170-017-0166-z 28261475 PMC5331634

[166] Giudetti AM De Domenico S Ragusa A Lunetti P Gaballo A Franck J. A specific lipid metabolic profile is associated with the epithelial mesenchymal transition program. Biochim Biophys Acta Mol Cell Biol Lipids. (2019) 1864:344–57. 10.1016/j.bbalip.2018.12.011 30578966

[167] Kuok IT Rountree AM Jung SR Sweet IR Palmitate is not an effective fuel for pancreatic islets and amplifies insulin secretion independent of calcium release from endoplasmic reticulum. Islets. (2019) 11:51–64. 10.1080/19382014.2019.1601490 31084524 PMC6548485

[168] Lehninger AL The Mitochondrion: Molecular Basis of Structure and Function. New York, NY: W.A. Benjamin, INC (1964).

[169] Samudio I Fiegl M Andreeff M. Mitochondrial uncoupling and the warburg effect: molecular basis for the reprogramming of cancer cell metabolism. Cancer Res. (2009) 69:2163–6. 10.1158/0008-5472.CAN-08-3722 19258498 PMC3822436

[170] Vozza A Parisi G De Leonardis F Lasorsa FM Castegna A Amorese D. UCP2 transports C4 metabolites out of mitochondria, regulating glucose and glutamine oxidation. Proc Natl Acad Sci USA. (2014) 111:960–5. 10.1073/pnas.1317400111 24395786 PMC3903233

[171] Hardy S El-Assaad W Przybytkowski E Joly E Prentki M Langelier Y. Saturated fatty acid-induced apoptosis in MDA-MB-231 breast cancer cells. a role for cardiolipin. J Biol Chem. (2003) 278:31861–70. 10.1074/jbc.M300190200 12805375

[172] Listenberger LL Han X Lewis SE Cases S Farese RV Jr Ory DS. Triglyceride accumulation protects against fatty acid-induced lipotoxicity. Proc Natl Acad Sci USA. (2003) 100:3077–82. 10.1073/pnas.0630588100 12629214 PMC152249

[173] Kamili A Roslan N Frost S Cantrill LC Wang D Della-Franca A. TPD52 expression increases neutral lipid storage within cultured cells. J Cell Sci. (2015) 128:3223–38. 10.1242/jcs.167692 26183179 PMC6518234

[174] Ta NL Seyfried TN. Influence of serum and hypoxia on incorporation of [(14)C]-D-glucose or [(14)C]-L-glutamine into lipids and lactate in murine glioblastoma cells. Lipids. (2015) 50:1167–84. 10.1007/s11745-015-4075-z 26537505

[175] Mahoney LB Denny CA Seyfried TN. Caloric restriction in C57BL/6J mice mimics therapeutic fasting in humans. Lipids Health Dis. (2006) 5:13. 10.1186/1476-511X-5-13 16709251 PMC1513228

[176] Meidenbauer JJ Ta N Seyfried TN. Influence of a ketogenic diet, fish-oil, and calorie restriction on plasma metabolites and lipids in C57BL/6J mice. Nutr Metab. (2014) 11:23. 10.1186/1743-7075-11-23 24910707 PMC4047269

[177] Kiebish MA Han X Cheng H Chuang JH Seyfried TN. Cardiolipin and electron transport chain abnormalities in mouse brain tumor mitochondria: lipidomic evidence supporting the Warburg theory of cancer. J Lipid Res. (2008) 49:2545–56. 10.1194/jlr.M800319-JLR200 18703489 PMC2582368

[178] Cogliati S Enriquez JA Scorrano L. Mitochondrial cristae: where beauty meets functionality. Trends Biochem Sci. (2016) 41:261–73. 10.1016/j.tibs.2016.01.001 26857402

[179] Leone RD Zhao L Englert JM Sun IM Oh MH Sun IH. Glutamine blockade induces divergent metabolic programs to overcome tumor immune evasion. Science. (2019) 366:1013–21. 10.1126/science.aav2588 31699883 PMC7023461

[180] Reckzeh ES Karageorgis G Schwalfenberg M Ceballos J Nowacki J Stroet MCM. Inhibition of glucose transporters and glutaminase synergistically impairs tumor cell growth. Cell Chem Biol. (2019) 26:1214–28 e1225. 10.1016/j.chembiol.2019.06.005 31303578

[181] Zhou W Mukherjee P Kiebish MA Markis WT Mantis JG Seyfried TN. The calorically restricted ketogenic diet, an effective alternative therapy for malignant brain cancer. Nutr Metab. (2007) 4:5. 10.1186/1743-7075-4-5 17313687 PMC1819381

[182] Mulrooney TJ Marsh J Urits I Seyfried TN Mukherjee P. Influence of Caloric Restriction on Constitutive Expression of NF-kappaB in an Experimental Mouse Astrocytoma. PLoS ONE. (2011) 6:e18085. 10.1371/journal.pone.0018085 21479220 PMC3068150

[183] Urits I Mukherjee P Meidenbauer J Seyfried TN Dietary restriction promotes vessel maturation in a mouse astrocytoma. J Oncol. (2012) 264039:10 10.1155/2012/264039 PMC3255299 22253625

[184] Mukherjee P El-Abbadi MM Kasperzyk JL Ranes MK Seyfried TN. Dietary restriction reduces angiogenesis and growth in an orthotopic mouse brain tumour model. Br J Cancer. (2002) 86:1615–21. 10.1038/sj.bjc.6600298 12085212 PMC2746602

[185] Mukherjee P Mulrooney TJ Marsh J Blair D Chiles TC Seyfried TN. Differential effects of energy stress on AMPK phosphorylation and apoptosis in experimental brain tumor and normal brain. Mol Cancer. (2008) 7:37. 10.1186/1476-4598-7-37 18474106 PMC2397440

[186] Shelton LM Huysentruyt LC Mukherjee P Seyfried TN. Calorie restriction as an anti-invasive therapy for malignant brain cancer in the VM mouse. ASN Neuro. (2010) 2:e00038. 10.1042/AN20100002 20664705 PMC2908744

[187] Simone BA Palagani A Strickland K Ko K Jin L Lim MK. Caloric restriction counteracts chemotherapy-induced inflammation and increases response to therapy in a triple negative breast cancer model. Cell Cycle. (2018) 17:1536–44. 10.1080/15384101.2018.1471314 29912618 PMC6133339

[188] Wallace TC Bultman S D'adamo C Daniel CR Debelius J Ho E. Personalized nutrition in disrupting cancer - proceedings from the 2017 american college of nutrition annual meeting. J Am Coll Nutr. (2019) 38:1–14. 10.1080/07315724.2018.1500499 30511901

[189] Veech RL. The therapeutic implications of ketone bodies: the effects of ketone bodies in pathological conditions: ketosis, ketogenic diet, redox states, insulin resistance, and mitochondrial metabolism. Prostaglandins Leukot Essent Fatty Acids. (2004) 70:309–19. 10.1016/j.plefa.2003.09.007 14769489

[190] D'agostino DP Olson JE Dean JB. Acute hyperoxia increases lipid peroxidation and induces plasma membrane blebbing in human U87 glioblastoma cells. Neuroscience. (2009) 159:1011–22. 10.1016/j.neuroscience.2009.01.062 19356685

[191] Veech RL Todd King M Pawlosky R Kashiwaya Y Bradshaw PC Curtis W. The great controlling nucleotide coenzymes. IUBMB Life. (2019) 71:565–79. 10.1002/iub.1997 30624851 PMC6850382

[192] Wu W Chaudhuri S Brickley DR Pang D Karrison T Conzen SD. Microarray analysis reveals glucocorticoid-regulated survival genes that are associated with inhibition of apoptosis in breast epithelial cells. Cancer Res. (2004) 64:1757–64. 10.1158/0008-5472.CAN-03-2546 14996737

[193] Champ CE Palmer JD Volek JS Werner-Wasik M Andrews DW Evans JJ. Targeting metabolism with a ketogenic diet during the treatment of glioblastoma multiforme. J Neuro Oncol. (2014) 117:125–31. 10.1007/s11060-014-1362-0 24442482

[194] Rieger J Bahr O Maurer GD Hattingen E Franz K Brucker D. ERGO: a pilot study of ketogenic diet in recurrent glioblastoma. Int J Oncol. (2014) 44:1843–52. 10.3892/ijo.2014.2382 24728273 PMC4063533

[195] De Beer JC Liebenberg L. Does cancer risk increase with HbA1c, independent of diabetes? Br J Cancer. (2014) 110:2361–8. 10.1038/bjc.2014.150 24675382 PMC4007234

[196] Wu K Yu X Huang Z Zhu D Yi X Wu YL. Targeting of PP2Cdelta By a small molecule C23 inhibits high glucose-induced breast cancer progression *in vivo*. Antioxid Redox Signal. (2019) 30:1983–98. 10.1089/ars.2017.7486 29808718 PMC6486665

[197] Meidenbauer JJ Mukherjee P Seyfried TN. The glucose ketone index calculator: a simple tool to monitor therapeutic efficacy for metabolic management of brain cancer. Nutr Metab. (2015) 12:12. 10.1186/s12986-015-0009-2 25798181 PMC4367849

[198] Emond JA Pierce JP Natarajan L Gapuz LR Nguyen J Parker BA. Risk of breast cancer recurrence associated with carbohydrate intake and tissue expression of IGFI receptor. Cancer Epidemiol Biomark Prev. (2014) 23:1273–9. 10.1158/1055-9965.EPI-13-1218 24755714 PMC4082443

[199] Klement RJ. Beneficial effects of ketogenic diets for cancer patients: a realist review with focus on evidence and confirmation. Med Oncol. (2017) 34:132. 10.1007/s12032-017-0991-5 28653283

[200] Santos JG Souza Da Cruz WM Schonthal AH Salazar MD Fontes CA Qiuirico-Santos T. Efficacy of a ketogenic diet with concomitant intranasal perillyl alcohol as a novel strategy for the therapy of recurrent glioblastoma. Onc Lett. (2018) 15:1263–70. 10.3892/ol.2017.7362 29391903 PMC5769394

[201] Poff AM Ari C Seyfried TN D'agostino DP. The ketogenic diet and hyperbaric oxygen therapy prolong survival in mice with systemic metastatic cancer. PLoS ONE. (2013) 8:e65522. 10.1371/journal.pone.0065522 23755243 PMC3673985

[202] Husain Z Huang Y Seth P Sukhatme VP. Tumor-derived lactate modifies antitumor immune response: effect on myeloid-derived suppressor cells and NK cells. J Immunol. (2013) 191:1486–95. 10.4049/jimmunol.1202702 23817426

[203] Denny CA Heinecke KA Kim YP Baek RC Loh KS Butters TD. Restricted ketogenic diet enhances the therapeutic action of N-butyldeoxynojirimycin towards brain GM2 accumulation in adult sandhoff disease mice. J Neurochem. (2010) 113:1525–35. 10.1111/j.1471-4159.2010.06733.x 20374428

[204] Al-Bari MA. Chloroquine analogues in drug discovery: new directions of uses, mechanisms of actions and toxic manifestations from malaria to multifarious diseases. J Antimicrob Chemother. (2015) 70:1608–21. 10.1093/jac/dkv018 25693996 PMC7537707

[205] Ye H Chen M Cao F Huang H Zhan R Zheng X. Chloroquine, an autophagy inhibitor, potentiates the radiosensitivity of glioma initiating cells by inhibiting autophagy and activating apoptosis. BMC Neurol. (2016) 16:178. 10.1186/s12883-016-0700-6 27644442 PMC5029068

[206] Hrabak A Sefrioui H Vercruysse V Temesi A Bajor T Vray B. Action of chloroquine on nitric oxide production and parasite killing by macrophages. Eur J Pharmacol. (1998) 354:83–90. 10.1016/S0014-2999(98)00427-0 9726634

[207] Yang C Ko B Hensley CT Jiang L Wasti AT Kim J. Glutamine oxidation maintains the TCA cycle and cell survival during impaired mitochondrial pyruvate transport. Mol Cell. (2014) 56:414–24. 10.1016/j.molcel.2014.09.025 25458842 PMC4268166

[208] Shelton LM Huysentruyt LC Seyfried TN. Glutamine targeting inhibits systemic metastasis in the VM-M3 murine tumor model. Inter J Cancer. (2010) 127:2478–85. 10.1002/ijc.25431 20473919 PMC2946425

[209] Lemberg KM Vornov JJ Rais R Slusher BS We're not DON Yet: optimal dosing and prodrug delivery of 6-Diazo-5-oxo-L-norleucine. Mol Cancer Ther. (2018) 17:1824–32. 10.1158/1535-7163.MCT-17-1148 30181331 PMC6130910

[210] Kalamian M KETO for CANCER: Ketogenic Metabolic Therapy as a Targeted Nutritional Strategy. White River Junction, VT: Chelsea Green (2017).

[211] Iyikesici MS. Feasibility study of metabolically supported chemotherapy with weekly carboplatin/paclitaxel combined with ketogenic diet, hyperthermia and hyperbaric oxygen therapy in metastatic non-small cell lung cancer. Int J Hyperthermia. (2019) 36:446–55. 10.1080/02656736.2019.1589584 30931666

[212] Elsakka AMA Bary MA Abdelzaher E Elnaggar M Kalamian M Mukherjee P. Management of glioblastoma multiforme in a patient treated with ketogenic metabolic therapy and modified standard of care: A 24-month follow-up. Front Nutr. (2018) 5:20. 10.3389/fnut.2018.00020 29651419 PMC5884883

[213] Arens NC West ID Press-pulse: a general theory of mass extinction? Paleobiology. (2008) 34:456–71. 10.1666/07034.1

[214] Mukherjee P Abate LE Seyfried TN. Antiangiogenic and proapoptotic effects of dietary restriction on experimental mouse and human brain tumors. Clin Cancer Res. (2004) 10:5622–9. 10.1158/1078-0432.CCR-04-0308 15328205

[215] Marsh J Mukherjee P Seyfried TN. Akt-dependent proapoptotic effects of dietary restriction on late-stage management of a phosphatase and tensin homologue/tuberous sclerosis complex 2-deficient mouse astrocytoma. Clin Cancer Res. (2008) 14:7751–62. 10.1158/1078-0432.CCR-08-0213 19047102

[216] Seyfried TN Mukherjee P. Targeting energy metabolism in brain cancer: review and hypothesis. Nutr Metab. (2005) 2:30. 10.1186/1743-7075-2-30 16242042 PMC1276814

